# Maternal and perinatal outcomes of prolonged second stage of labour: a historical cohort study of over 51,000 women

**DOI:** 10.1186/s12884-023-05733-z

**Published:** 2023-06-22

**Authors:** Catriona Young, Sohinee Bhattacharya, Andrea Woolner, Amy Ingram, Nicole Smith, Edwin-Amalraj Raja, Mairead Black

**Affiliations:** 1 Aberdeen Centre for Women’s Health Research (ACWHR), Foresterhill, University of Aberdeen, Aberdeen, AB25 2ZD UK; 2 Raigmore Hospital, NHS Highland, Inverness, IV2 3UJ UK; 3 Golden Jubilee Hospital, NHS Greater Glasgow and Clyde, Clydebank, G81 4DY UK; 4 School of Medicine, University of Aberdeen, Aberdeen, AB25 2ZD UK

**Keywords:** Second stage, Birth, Prolonged labour

## Abstract

**Background:**

Prolonged second stage of labour has been associated with adverse maternal and perinatal outcomes. The maximum length of the second stage from full dilatation to birth of the baby remains controversial. Our aim was to determine whether extending second stage of labour was associated with adverse maternal and perinatal outcomes.

**Methods:**

A retrospective cohort study was conducted using routinely collected hospital data from 51592 births in Aberdeen Maternity Hospital between 2000 and 2016. The hospital followed the local guidance of allowing second stage of labour to extend by an hour compared to national guidelines since 2008 (nulliparous and parous). The increasing duration of second stage of labour was the exposure. Baseline characteristics, maternal and perinatal outcomes were compared between women who had a second stage labour of (a) ≤ 3 h and (b) > 3 h duration for nulliparous women; and (a) ≤ 2 h or (b) > 2 h for parous women. An additional model was run that treated the duration of second stage of labour as a continuous variable (measured in hours). All the adjusted models accounted for: age, BMI, smoking status, deprivation category, induced birth, epidural, oxytocin, gestational age, baby birthweight, mode of birth and parity (only for the final model).

**Results:**

Each hourly increase in the second stage of labour was associated with an increased risk of obstetric anal sphincter injury (aOR 1.21 95% CI 1.16,1.25), having an episiotomy (aOR 1.48 95% CI 1.45, 1.52) and postpartum haemorrhage (aOR 1.27 95% CI 1.25, 1.30). The rates of caesarean and forceps delivery also increased when second stage duration increased (aOR 2.60 95% CI 2.50, 2.70, and aOR 2.44 95% CI 2.38, 2.51, respectively.) Overall adverse perinatal outcomes were not found to change significantly with duration of second stage on multivariate analysis.

**Conclusions:**

As the duration of second stage of labour increased each hour, the risk of obstetric anal sphincter injuries, episiotomies and PPH increases significantly. Women were over 2 times more likely to have a forceps or caesarean birth. The association between adverse perinatal outcomes and the duration of second stage of labour was less convincing in this study.

**Supplementary Information:**

The online version contains supplementary material available at 10.1186/s12884-023-05733-z.

## Introduction

The second stage of labour varies in duration from minutes to many hours. Prolonged second stage, particularly where labour is obstructed, has been associated with complications such as Postpartum Haemorrhage (PPH) and in severe cases obstetric fistula. However, the optimal limit on the duration of second stage, in the absence of fetal concerns or maternal exhaustion, after which an intervention to expedite the birth should be recommended is not known.

A decision to intervene to limit the second stage of labour requires a balance of the risks and benefits compared to awaiting spontaneous birth. Operative vaginal birth risks include Obstetric Anal Sphincter Injury (OASI) and rarely nerve or bony injury for the newborn [1]. Second stage caesarean birth also presents a substantial risk of maternal trauma, haemorrhage and risk of trauma to the infant [2], as well as the risks for subsequent pregnancies. Both interventions are also linked to maternal post-traumatic stress symptoms [3]. Some mothers may wish to continue to try for a Spontaneous Vaginal Delivery (SVD) if they believe the likelihood increases over time and as such considers it a perceived benefit.

While the evidence is limited, the 2017 National Institute for Health and Care Excellence (NICE) has recommended offering intervention after 3 h of active second stage for nulliparous and 2 h for parous women [4]. Prior to this, a 2006 systematic review [5] demonstrated a lack of reliable evidence that the duration of second stage was linked to adverse maternal or perinatal outcomes. Study limitations at the time included broad categorisations of second stage duration (i.e. < 3 h vs. > 3 h) and lack of data on potential confounding factors. However, the 2006 review led to changes in clinical guidance in Aberdeen (NHS Grampian) which involved offering intervention after 4 h of second stage for nulliparous and 3 h for parous women, taking effect in 2008. The Aberdeen Maternity and Neonatal Databank (AMND) includes detailed labour stage duration data along with multiple covariates and captures sufficient cases exceeding 4 h in second stage to enable this detailed study of prolonged second stage and the associated risks. The AMND had the unique opportunity to analyse prolonged second stage due to this change in local guidance.

The aim of this study was to determine whether extending second stage of labour was associated with adverse maternal and perinatal outcomes.

## Methods

### Design

This study looks at the relationship between the duration of second stage of labour and adverse outcomes for mothers and newborns. The findings of this study are key in providing women and clinicians with information to support informed decisions on whether and when to intervene in prolonged second stage of labour with an increased awareness of the heightened risk.

A population-based retrospective cohort study using routinely collected anonymised data was conducted to determine whether increasing the duration of second stage of labour was associated with a corresponding increase in the risks of morbidities for mother and newborn. The population studied included all singleton births at or beyond 37 weeks gestation reaching second stage of labour that occurred between 2000 and 2016 inclusive. Exclusion criteria referred to elective caesarean sections, before 37 weeks gestation, known intrauterine deaths, vaginal breech births and multiple gestations.

This study data source was the AMND [6] which includes routinely collected data for all pregnancies and babies where birth occurred in Aberdeen City and District since 1950. This means AMND captures births prior to a change in local policy on second stage management before 2008 (length of labour similar to the rest of the country) in addition to those with a significantly prolonged second stage of labour (predominantly after 2008).

Study approval was gained from the AMND steering committee, which has ethical approval from the North of Scotland Research Ethics Service for studies which utilises anonymised unlinked data (19/NS/0079). The data was stored on a secure password protected University server. Regular quality assurance checks of AMND data were carried out [6].

Pregnancies were excluded if there was a known intrauterine death as data did not allow for differentiation between antepartum and intrapartum stillbirth. Furthermore, labour and birth outcomes are likely to be significantly different for stillborn babies. Vaginal breech births were also excluded.

The services captured by the databank follows the national guidelines on fetal monitoring by using intermittent auscultation in low-risk women and higher risk women would receive Cardiotocography (CTG) and labour ward care. When the second stage of labour became prolonged the monitoring would switch from intermittent auscultation to CTG.

### Data management

The data was cleaned by removing cases with inconceivable values for the following characteristics and outcomes: duration of second stage of labour, baby birthweight, cord pH and length of stay. A complete case analysis was performed.

Maternal outcome data included mode of birth, episiotomy, OASI, and PPH. Perinatal outcome data included Apgar score < 7 at 5 min, cord pH and admission to Neonatal Intensive Care Unit (NICU). Additional variables obtained included the duration of second stage of labour (exposure), parity and clinical and demographic characteristics that could be potential confounders (Tables 1 and 2 and Additional file 1: Appendix 1).

**Table 1:** Characteristics between < 3 versus ≥ 3 h 2^nd^ stage of labour (nulliparous women)

		Duration of second stage of labour				
		< 3 h		≥ 3 h		*P*-value
**Characteristics**		n/x̅/M^a^	%/SD/IQR^b^	n/x̅M	%/SD/IQR	
Age ( years)		27	(6)	29	(5)	** < 0.01**
BMI^c^	Underweight	699	(3.8%)	147	(2.5%)	** < 0.01**
	Healthy weight	10,553	(56.7%)	3205	(55.5%)	
	Overweight	4834	(26.0%)	1604	(27.8%)	
	Obese	2523	(13.6%)	815	(14.1%)	
Patient’s smoking status	Non-smoker	15,558	(82.1%)	5351	(90.8%)	** < 0.01**
	Smoker	3400	(17.9%)	543	(9.2%)	
Deprivation category^d^	1	3560	(19.1%)	1287	(22.5%)	** < 0.01**
	2	5294	(28.3%)	1725	(30.1%)	
	3	3291	(17.6%)	1012	(17.7%)	
	4	3534	(18.9%)	1031	(18.0%)	
	5	1049	(5.6%)	264	(4.6%)	
	6	1958	(10.5%)	407	(7.1%)	
Preeclampsia and other hypertensive disease	None	16,343	(84.5%)	4827	(81.1%)	**0.03**
	Pre-/eclampsia	907	(4.7%)	320	(5.4%)	
	Others forms	2089	(10.8%)	803	(13.5%)	
Type of labour	Spontaneous	13,202	(68.3%)	3920	(65.9%)	** < 0.01**
	Induced	6137	(31.7%)	2030	(34.1%)	
Epidural	Yes	4947	(25.6%)	3005	(50.5%)	** < 0.01**
	No	14,392	(74.4%)	2945	(49.5%)	
Oxytocin	Yes	3068	(15.9%)	1120	(18.8%)	** < 0.01**
	No	16,271	(84.1%)	4830	(81.2%)	
Antepartum haemorrhage	None	16,928	(87.5%)	5267	(88.5%)	**0.07**
	Abruption	48	(0.2%)	5	(0.1%)	
	Placenta previa	0	(0.0%)	1	(0.0%)	
	Unspecified	2363	(12.2%)	677	(11.4%)	
Gestational age	37	1027	(5.3%)	217	(3.6%)	** < 0.01**
	38	2128	(11.0%)	510	(8.6%)	
	39	4198	(21.7%)	1130	(19.0%)	
	40	6260	(32.4%)	1984	(33.3%)	
	41	5076	(26.2%)	1777	(29.9%)	
	42	650	(3.4%)	332	(5.6%)	
Baby birthweight		3366	(453)	3561	(432)	** < 0.01**
Years relative to change local guidance	2000–2007	9622	(49.8%)	1513	(25.4%)	
	2008–2016	9717	(50.2%)	4437	(74.6%)	^e^

^a^n/x̅/M, count/mean/median

^b^The values in brackets represents the percentage of the characteristic/outcome at that time interval. There are the exceptions of age, baby birthweight, and cord pH which shows their mean accompanied by the standard deviation in brackets and length of stay which has its median accompanied by the interquartile range. Statistically significant p values are shown as bold

^c^Body mass index

^d^Carstairs and Morris Index of deprivation

^e^This does not act as a potential cofounder, rather it highlights that despite the impact of the local guidance causing more > 3 h second stages of labour, some prolonged labour occurred prior to 2008

**Table 2:** Characteristics between < 2 versus ≥ 2 h 2^nd^ stage of labour (parous women)

		Duration of second stage of labour				
		< 2 h		≥ 2 h		*P*-value
**Characteristics**		n	%/SD/IQR^ab^	n	%/SD/IQR^ab^	
Age		31	(5)	32	(5)	**0.02**
BMI^c^	Underweight	524	(2.1%)	9	(1.1%)	0.41
	Healthy weight	12,232	(49.7%)	410	(49.5%)	
	Overweight	7247	(29.5%)	277	(33.5%)	
	Obese	4596	(18.7%)	132	(15.9%)	
Patient’s smoking status	Non-smoker	19,355	(81.1%)	1688	(88.8%)	** < 0.01**
	Smoker	4507	(18.9%)	213	(11.2%)	
Deprivation category^d^	1	5500	(23.4%)	475	(25.7%)	** < 0.01**
	2	6765	(28.7%)	545	(29.5%)	
	3	3754	(15.9%)	340	(18.4%)	
	4	3842	(16.3%)	277	(15.0%)	
	5	1129	(4.8%)	81	(4.4%)	
	6	2548	(10.8%)	131	(7.1%)	
Preeclampsia and other hypertensive disease	None	22,568	(92.5%)	1732	(89.3%)	**0.10**
	Pre-/eclampsia	407	(1.7%)	47	(8.3%)	
	Others forms	1396	(5.7%)	161	(2.4%)	
Type of labour	Spontaneous	17,803	(73.1%)	1309	(67.5%)	** < 0.01**
	Induced	6567	(26.9%)	631	(32.5%)	
Epidural	Yes	1844	(7.6%)	1029	(53.0%)	** < 0.01**
	No	22,526	(92.4%)	911	(47.0%)	
Oxytocin	Yes	1606	(6.6%)	183	(9.4%)	**0.04**
	No	22,764	(93.4%)	1757	(90.6%)	
Antepartum haemorrhage	None	22,017	(90.3%)	1743	(89.8%)	0.53
	Abruption	59	(0.2%)	7	(0.4%)	
	Placenta previa	2	(0.0%)	0	(0.0%)	
	Unspecified	2293	(9.4%)	190	(9.8%)	
Gestational age	37	1208	(5.0%)	32	(3.7%)	** < 0.01**
	38	2843	(11.7%)	84	(9.8%)	
	39	5812	(23.8%)	190	(22.1%)	
	40	8419	(34.4%)	282	(32.9%)	
	41	5658	(23.3%)	246	(28.7%)	
	42	431	(1.8%)	24	(2.8%)	
Baby birthweight		3535	(480)	3678	(475)	0.46
Years relative to change local guidance	2000–2007	11,422	(46.9%)	750	(38.7%)	
	2008–2016	12,948	(53.1%)	1190	(61.3%)	^e^

^a^n/x̅/M, count/mean/median

^b^The values in brackets represents the percentage of the characteristic/outcome at that time interval. There are the exceptions of age, baby birthweight, and cord pH which shows their mean accompanied by the standard deviation in brackets and length of stay which has its median accompanied by the interquartile range. Statistically significant p-values are shown as bold

^c^Body mass index

^d^Carstairs and Morris Index of deprivation

^e^This does not act as a potential cofounder, rather it highlights that despite the impact of the local guidance causing more > 3 h second stages of labour, some prolonged labour occurred prior to 2008

### Data analysis

Potential confounders were identified by comparing the characteristics of those nulliparous women who had a second stage of labour lasting < 3 versus > 3 h and parous < 2 versus > 2 h. These intervals were coded as 0–119 min for < 2 h and 120 to maximum (8 h) for > 2 h, and the equivalent methodology for < 3 (0–179 min) and > 3 h (180–480 min). Statistical analysis was carried out using Chi-squared test, independent t-test and one way ANOVA according to variable type. Only variables that were found to be significant (p < 0.05) were included in the multivariate analysis.

The outcomes were initially compared in the dichotomous manner for the nulliparous and parous cohorts separately using binary logistic regression and multinomial regression for mode of birth across the appropriate cut-off point for that cohort (Tables 3 and 4).

**Table 3:** Outcomes between < 3 versus ≥ 3 h 2^nd^ stage of labour (nulliparous women)

		Unadjusted model		Adjusted model	
Outcomes		OR^a^	95% CI^b^	Aor^c^	95% CI^b^
OASI^d^		**1.91**	**(1.72, 2.12)**	**1.57**	**(1.37, 1.79)**
Episiotomy		**2.69**	**(2.54, 2.86**	**2.35**	**(2.17, 2.54)**
Mode of birth	SVD (ref.^e^)				
	Forceps	**12.48**	**(11.55, 13.49)**	**9.53**	**(8.71, 10.41)**
	Caesarean	**18.36**	**(16.15, 20.86)**	**13.24**	**(11.50, 15.24)**
PPH^f^		**2.93**	**(2.75. 3.11)**	**1.95**	**(1.80, 2.12)**
Admission to NICU^g^		1.04	(0.95, 1.14)	0.87	(0.78, 0.99)
APGAR^h^ < 7 at 5 min		0.96	(0.72, 1.28)	0.84	(0.59, 1.20)
Cord Ph		0.01	(0.01, 0.01)	^i^	

^a^Odds ratio

^b^95% confidence intervals

^c^Adjusted odds ratio

^d^Obstetric anal sphincter injury, referring to muscular disruption of the sphincter muscles during childbirth which encapsulates 3^rd^ and 4^th^ degree tears [9, 10]

^e^SVD is the reference category for the regression model

^f^PPH is defined as > 500 ml for SVD and > 1000mls for caesarean section [7]

^g^Admission to neonatal intensive care

^h^Appearance, Pulse, Grimace, Activity, and Respiration

^i^Insufficient numbers of cases to perform multivariate analysis

**Table 4:** Outcomes between < 2 versus ≥ 2 h 2^nd^ stage of labour (parous women)

		Unadjusted model		Adjusted model	
Outcomes		OR^a^	95% CI^b^	aOR^c^	95% CI^b^
OASI^d^		**2.93**	**(2.29, 3.75)**	**1.98**	**(1.40, 2.81)**
Episiotomy		**9.15**	**(8.24, 10.17)**	**4.45**	**(3.82, 5.19)**
Mode of birth	SVD (ref.^e^)				
	Forceps	**41.99**	**(36.57, 48.21)**	**22.50**	**(19.12, 26.48)**
	Caesarean	**42.35**	**(33.20, 54.03)**	**28.24**	**(21.31, 37.43)**
PPH^f^		**3.95**	**(3.55, 4.39)**	**2.13**	**(1.83, 2.49)**
Admission to NICU^g^		1.27	(1.09, 1.49)	0.90	(0.72, 1.12)
APGAR < 7^ h^ at 5 min		**1.66**	**(1.08, 2.55)**	1.15	(0.65, 2.03)
Cord pH		0.00	(-0.01, 0.01)	^i^	

^a^Odds ratio

^b^95% confidence intervals

^c^Adjusted odds ratio

^d^Obstetric anal sphincter injury, referring to muscular disruption of the sphincter muscles during childbirth which encapsulates 3rd and 4th degree tears [9, 10]

^e^SVD is the reference category for the regression model

^f^PPH is defined as > 500 ml for SVD and > 1000mls for caesarean section [7]

^g^Admission to neonatal intensive care

^h^Appearance, Pulse, Grimace, Activity, and Respiration

^i^Insufficient numbers of cases to perform multivariate analysis

A model was then created to treat the duration of second stage of labour as a continuous variable measured in hours and combined the nulliparous and parous cohorts for this analysis (Table 5). Outcomes included were OASI, episiotomy, PPH, mode of birth and admission to NICU. PPH was defined as > 500 ml for SVD and > 1000 ml for caesarean section [7]. Cord pH was not included in the models due to high volume of missing data since it is measured only when clinically indicated. The following models were used for the analysis: binary logistic regression for binary outcomes and multinomial regression for categorical variables.

**Table 5:** Unadjusted and adjusted Odds ratios of adverse maternal and perinatal outcomes for hourly increase in the duration of the second stage of labour (continuous variable, measured in hours)

		Unadjusted Model		Adjusted Model^a^	
		OR^b^	95% CI^c^	OR^b^	95% CI^c^
Maternal outcomes					
OASI^d^		**1.43**	**(1.40, 1.47)**	**1.21**	**(1.16, 1.25)**
Episiotomy		**1.84**	**(1.81, 1.87)**	**1.48**	**(1.45, 1.52)**
Mode of birth	SVD (ref.)^e^	1.00		1.00	
	Forceps	**3.19**	**(3.11, 3.26)**	**2.44**	**(2.38, 2.51)**
	Caesarean	**3.37**	**(3.26, 3.49)**	**2.60**	**(2.50, 2.70)**
PPH^f^		**1.55**	**(1.53, 1.58)**	**1.27**	**(1.25, 1.30)**
Perinatal outcomes					
Admission to NICU^g^		**1.06**	**(1.04, 1.08)**	**0.96**	**(0.93, 0.98)**

^a^adjusted for parity, age, BMI, smoking status, deprivation category, spontaneous/induced labour, epidural, oxytocin use, gestational age and baby birthweight

^b^Odds ratio

^c^95% confidence intervals

^d^ *OASI* Obstetric Anal Sphincter Injury, referring to muscular disruption of the sphincter muscles during childbirth which encapsulates 3rd and 4th degree tears [9, 10]

^e^ *SVD* Spontaneous vaginal birth, is the reference category

^f^PPH is defined as > 500 ml for SVD and > 1000mls for caesarean section [7]

^g^Admission to neonatal intensive care unit

Statistically significant odds ratios are shown as bold

The counts and proportions of the adverse outcomes were also evaluated as they changed over each hour increment (at a maximum of 8 h), see Additional file 1: Appendix 2 and 3 *.*

## Results

The 2000–2016 cohort included 51,592 women reaching the second stage of labour after removing the 172 cases with inconceivable values, 49 cases due to known intrauterine death and 94 cases of breech deliveries. In total 25,282 women were nulliparous and 26,310 were parous (Additional file 1: Appendix 4). Among mothers recorded, 87% delivered before 3 h of second stage (93% among parous women and 76% among nulliparous women). There were 1409 instances of second stage of labour that exceeded 5 h. There were 1818 missing cases from the original database (3.4%).

Several characteristics significantly differed between those delivering before 2 or 3 h compared to those with prolonged labour. These characteristics were age, BMI, patient’s smoking status, deprivation category (The Carstairs and Morris Index) [8], type of labour, epidural, oxytocin use, gestational age and baby birthweight (Tables 1 and 2). As a result, all of these were included as potential confounders in the multivariate analysis, alongside mode of birth.

### Maternal outcomes

In the nulliparous cohort, there was an increased odds of 1.57 for OASI after 3 h (aOR 1.57 95% CI 1.37, 1.79). While parous mothers had an increased odds of 1.98 (aOR 1.98 95% CI 1.40, 2.81) after 2 h (Tables 3 and 4*).* The odds ratio for OASI was significant for both the unadjusted and adjusted models. In the nulliparous cohort, there was an increased odds of 1.95 for PPH after 3 h (aOR 1.95 95% CI 1.80, 2.12). While parous mothers had an increased odds of 2.13 for PPH after 3 h (aOR 2.13 95% CI 1.83, 2.49). The odds for both forceps and caesarean sections increased significantly in both cohorts once second stage of labour surpassed the national guidance.

The model that used second stage of labour as a continuous variable (Table 5) demonstrated a significant odds ratio for OASI (OR 1.43 95% CI 1.40, 1.47 and aOR 1.21 95% CI 1.16, 1.25). The odds for episiotomy also increased once beyond the national guidance (OR 1.84 95% CI 1.81,1.87 and aOR 1.48 95% CI 1.45, 1.52). The odds of episiotomy increased by 48% for every hour that the second stage of labour lasted, see Table 5.

The same models (Table 5) indicated significant ORs for forceps in the unadjusted (OR 3.19 95% CI 3.11, 3.26) and the adjusted (aOR 2.44 95% CI 2.38, 2.51) models. The association was also significant for caesarean sections (OR 3.37 95% CI 3.26, 3.49 and aOR 2.60 95% CI 2.50, 2.70).

The odds of PPH increased significantly as second stage of labour extended indicated by the significant ORs (Table 5) (OR 1.55 95% CI 1.53, 1.58 and aOR 1.27 95% CI 1.25, 1.30).

In Additional file 1: Appendix 2 and 3, which shows the number and proportion of outcomes at each hour increment after the cut-off (2 or 3 h according to the cohort), suggests the proportion of forceps continues to grow as the second stage of labour prolongs. Whereas caesarean sections jump from 2.5% at < 3 h to 15.2% at 3–4 h in the nulliparous cohort and remains around this figure even at 4–5 h (15.2%). A similar trend of forceps and caesarean sections is observed in the parous cohort.

### Perinatal outcomes

In Tables 3 and 4, the associations between the duration second stage of labour and adverse perinatal outcomes were not significant in the multivariate analysis, with the exception of admission to NICU in the nulliparous cohort (aOR 0.87 95% CI 0.78, 0.99). Once duration was treated as a continuous variable, Table 5, significant associations between the duration of second stage of labour and adverse perinatal outcomes were not identified in this direction in the adjusted models. Rather this model indicated a reduced odds of admission to NICU when second stage extends (aOR 0.96 95% CI 0.93, 0.98).

## Discussion

### Main findings

This large cohort study of an entire obstetric population demonstrated a significant increase in risk of OASI, episiotomies and PPH with longer second stage of labour (Table 5). These heightened risks identified once second stage surpasses the established NICE guidance [4], provides evidence supporting these guidelines (Table 3 and 4). As second stage of labour duration increased, the number of caesarean sections and forceps deliveries also increased while SVD fell in both cohorts. A significant association between the duration of second stage of labour and adverse perinatal outcomes for the most part was not identified. However, after adjusting for confounding factors, the odds of NICU admission changed direction, such that with increasing duration of 2nd stage, the odds of NICU admission were reduced.

### Findings in the context of existing literature

A 2006 systematic review [5] did not establish an optimal duration of second stage of labour, partly citing the dichotomous approach to second stage duration (< vs. > a certain duration [11–13] in most studies), as well as insufficient adjustment of confounders [13, 14].

More recently, large retrospective cohort studies have reported that prolonged second stage of labour is linked to chorioamnionitis and OASI [15, 16], alongside birth asphyxia and admission to NICU [15–17]. The increased risk of NICU admission has been previously reported [14], alongside APGAR scores < 7 [18]. A secondary analysis of trials with women pushing with an epidural found PPH and intrapartum fever increased with longer durations as well as the increased risk of asphyxia [19], while other trials did not identify the same risk for newborns [20, 21].

One such trial among nulliparous mothers receiving epidural analgesia showed prolonged labour was linked to lower rates of caesarean sections [21]. This trial is supported by further studies [22, 23]. However, our study is not alone in showing an increase of operative births as second stage duration extends [15, 24].

The findings of this study support the earlier evidence which highlights an increased risk of PPH and OASI without an increased risk to perinatal outcomes as the duration of second stage increases. The lack of statistically significant association between duration of second stage of labour and adverse perinatal outcomes in this study may be explained by any indication of fetal compromise as duration of second stage increases being more likely to prompt the decision to deliver by caesarean or forceps-assisted birth.

### Clinical implications

**Fig. 1:**
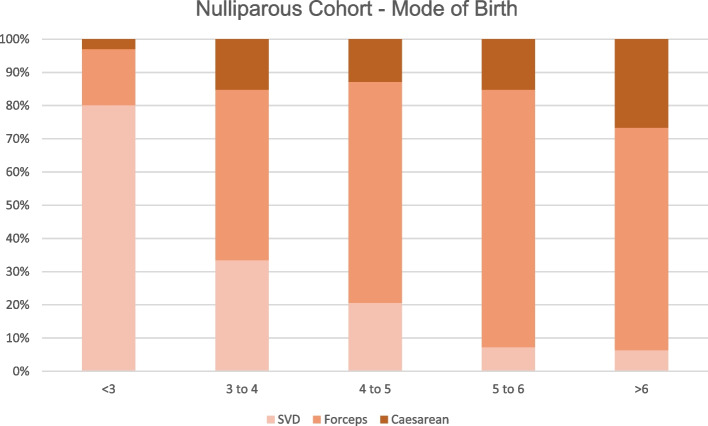
Comparison of mode of birth through increasing hour increments of second stage of labour in the nulliparous cohort

**Fig. 2:**
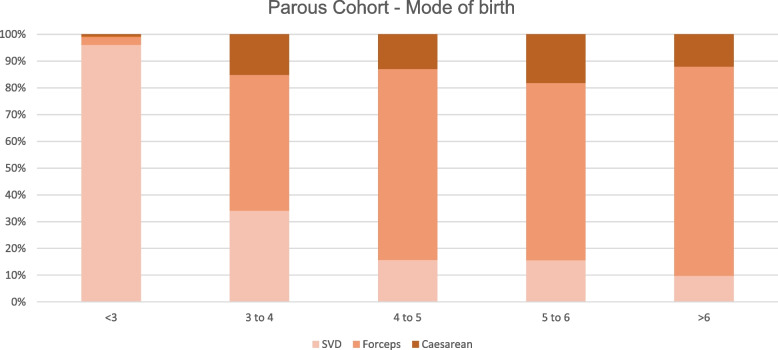
Comparison of mode of birth through increasing hour increments of second stage of labour in the parous cohort

As the duration of second stage increased, forceps and episiotomies became more likely. Many feel the perceptions of forceps and episiotomies have worsened with the public and that caesareans are more acceptable such that these findings have the potential to influence earlier intervention by Caesarean [25]. The changing picture of mode of birth as the second stage of labour extends is visually represented by Figs. 1 and 2. The increased risk of PPH and OASI was also significant. As such there is a need to address this issue in nulliparous antenatal counselling by exploring their wishes in a scenario of prolonged second stage of labour.

### Strengths and weaknesses

In total, the cohort captured 1409 births that had a second stage of labour 5 h or longer. The opportunity to study very prolonged (> 300 min [26]) second stage of labour has facilitated a deeper understanding of how the duration impacts outcomes. The data is examined by using the cut-off set by national guidance [4] as well as analysing the risk as duration increases hourly.

The large cohort which captured 51,592 births allowed for an analysis that accounted for multiple potential confounders, including mode of birth. In the 2006 review, the average population size under review was dramatically smaller than the AMND cohort at 7354, with the largest of them having 25,069 mothers [27].

The AMND created a unique opportunity for this analysis due to the local guideline changes.

The duration of labour across the cohort was heavily populated with mothers delivering before 3 h. This is especially true for the parous women (24,370/26310 = 93%). It means the cohorts of women delivering > 3 h (7% in parous women) does not have the same level of evidence. However, the study was sufficiently powered to allow for this uneven distribution in most parameters.

This study adjusted the analyses for extensive potential confounders. However, regional practice and norms influence duration of second stage of labour, mode of birth and episiotomies. This may limit the application of results which is a shared obstacle for all studies on this topic and may explain the difference in findings to other studies [21–23] that followed mode of birth according to duration. The study also does not provide all the clinical indications that would influence the duration of second stage of labour. These women still go through the second stage of labour (elective caesareans already excluded) and contribute to the understanding of how duration impacts outcomes. Pre-eclampsia/eclampsia has been adjusted for in the analysis while all potential indications for intervening is beyond the remit of the research question.

Other studies split the cohort between those receiving an epidural or not [23, 28, 29]. Although epidurals may influence both the duration and mode of birth, they were adjusted for in the multivariate analysis. The split by nulliparous/parous appears more appropriate since this is known before going into labour and cannot be changed compared to epidurals.

## Conclusion

Prolonged second stage of labour is associated with a significantly increased risk of OASI, having an episiotomy, PPH, forceps-assisted birth and caesarean sections. The association of increasing duration of second stage with adverse perinatal outcomes were less clear. Subsequently, low-risk mothers may wish to prolong the second stage beyond the current national guidance if prioritising a vaginal birth as long as other parameters remain normal.

## Supplementary Information

**Additional file1: Appendix 1. **Directedacyclic graph of the exposures, confounders and outcomes in the study. **Appendix 2. **Count and proportion of adverse outcomes by hourly intervals of 2^nd^ stage duration in nulliparous women. **Appendix 3.** Count and proportion of adverse outcomes by hourly intervals of 2^nd^ stage duration in parous women. **Appendix 4.** Flowchart of the population included in the study. 

## Data Availability

The datasets used and analysed during the current study are available from the corresponding author on reasonable request via an application to the steering committee of the Aberdeen Maternity and Neonatal Databank. There is a financial cost for the service..

## Abbreviations

PPH: Postpartum Haemorrhage
OASI: Obstetric Anal Sphincter Injury
AMND: Aberdeen Maternity and Neonatal Databank
NICU: Neonatal Intensive Care Unit
OR: Odds Ratio
CI: Confidence Intervals
SVDs: Spontaneous Vaginal Deliveries
CTG: Cardiotocography
