# Supplementary material for: Maternal and perinatal outcomes of prolonged second stage of labour: a historical cohort study of over 51,000 women
Source: BMC Pregnancy Childbirth. 2023 Jun 22;23:467. doi: 10.1186/s12884-023-05733-z (PMC10288707; doi:10.1186/s12884-023-05733-z)

Appendix 1 - Directed acyclic graph of the exposure, confounders and outcomes in the study.


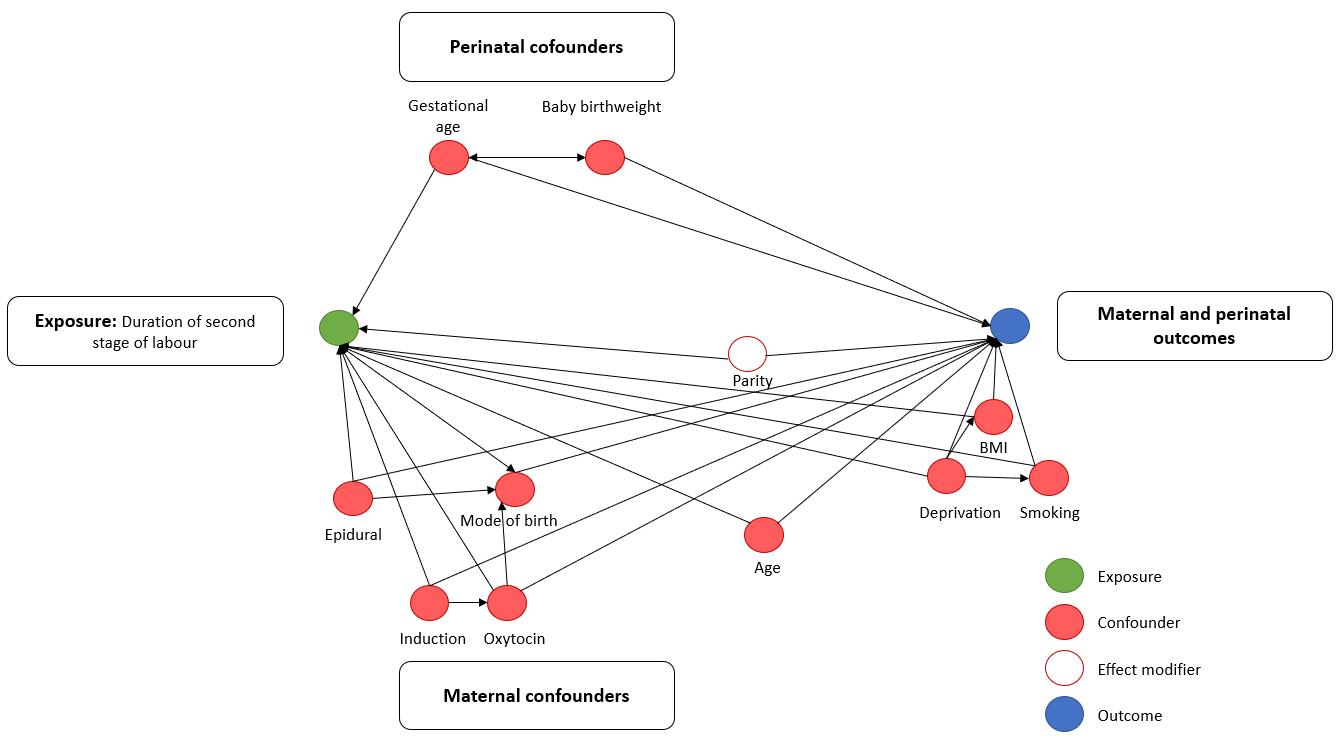


Appendix 2 – Count and proportion of adverse outcomes by hourly intervals of 2^nd^ stage duration in nulliparous women

|  |  | 0-179 minutes  (n=19339) | | 180-239 minutes  (n=2898) | | 240-299  minutes  (n=1749) | | 300-359 minutes  (n=950) | | >359 minutes  (n=346) | |
| --- | --- | --- | --- | --- | --- | --- | --- | --- | --- | --- | --- |
|  |  | n | % | n | % | n | % | n | % | n | % |
| OASI |  | 1078 | 5.6 | 237 | 8.2 | 198 | 11.3 | 122 | 12.8 | 46 | 13.3 |
| Episiotomy |  | 5552 | 28.7 | 1439 | 49.7 | 968 | 55.3 | 526 | 55.4 | 183 | 47.1 |
| Mode of birth^a^ | SVD | 13282 | 68.7 | 803 | 33.5 | 319 | 20.6 | 66 | 7.3 | 21 | 6.4 |
|  | Forceps | 2800 | 14.5 | 1228 | 51.3 | 1033 | 66.6 | 695 | 77.5 | 221 | 66.7 |
|  | Caesarean | 474 | 2.5 | 365 | 15.2 | 200 | 12.9 | 137 | 15.2 | 88 | 26.7 |
| PPH |  | 3794 | 19.6 | 1015 | 35.0 | 810 | 46.3 | 500 | 52.6 | 155 | 44.8 |
| Admission to NICU |  | 2009 | 10.4 | 64 | 12.4 | 17 | 8.0 | 9 | 11.4 | 3 | 9.7 |

^a^Vaccum extractions (n=2783, 14.4%) were not included in the regression models.

Appendix 3 – Count and proportion of adverse outcomes by hourly intervals of 2^nd^ stage duration in parous women

|  |  | 0-119 minutes  (n=24370) | | 120-179 minutes  (n=1082) | | 180-239  minutes  (n=530) | | 240-299  minutes  (n=214) | | 300-359  minutes  (n=82) | | >359 minutes  (n=32) | |
| --- | --- | --- | --- | --- | --- | --- | --- | --- | --- | --- | --- | --- | --- |
|  |  | n | % | n | % | n | % | n | % | n | % | n | % |
| OASI |  | 358 | 1.5 | 38 | 3.5 | 20 | 3.8 | 13 | 6.1 | 6 | 7.3 | 4 | 12.5 |
| Episiotomy |  | 1541 | 6.3 | 340 | 31.4 | 225 | 42.5 | 113 | 52.8 | 44 | 53.7 | 17 | 53.1 |
| Mode of birth^a^ | SVD | 23100 | 94.8 | 558 | 66.2 | 158 | 35.3 | 29 | 15.3 | 12 | 15.6 | 3 | 9.4 |
|  | Forceps | 478 | 2.0 | 226 | 26.8 | 221 | 49.3 | 136 | 72.0 | 51 | 66.2 | 25 | 78.1 |
|  | Caesarean | 122 | 0.5 | 59 | 7.0 | 69 | 15.4 | 24 | 12.7 | 14 | 18.2 | 4 | 12.5 |
| PPH |  | 2286 | 9.4 | 257 | 23.8 | 173 | 32.6 | 83 | 38.8 | 39 | 47.6 | 11 | 34.4 |
| Admission to NICU |  | 1926 | 7.9 | 99 | 9.2 | 65 | 12.3 | 17 | 7.9 | 8 | 10.0 | 4 | 12.5 |

^a^Vaccum extractions (n=670, 2.7%) were not included in the regression models.

Appendix 4 - Flowchart of the population included in the study


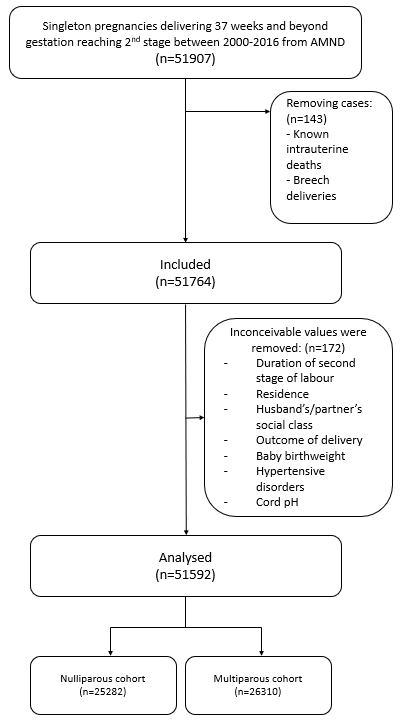

Supplement: Supplementary file 1 — Additional file1: Appendix 1. Directedacyclic graph of the exposures, confounders and outcomes in the study. Appendix 2. Count and proportion of adverse outcomes by hourly intervals of 2nd stage duration in nulliparous women. Appendix 3. Count and proportion of adverse outcomes by hourly intervals of 2nd stage duration in parous women. Appendix 4. Flowchart of the population included in the study. [file 12884_2023_5733_MOESM1_ESM.docx]
